# How membrane structures control T cell signaling.

**DOI:** 10.3389/fimmu.2012.00291

**Published:** 2012-09-21

**Authors:** Christian Klammt, Björn F. Lillemeier

**Affiliations:** Nomis Center for Immunobiology and Microbial Pathogenesis, Waitt Advanced Biophotonics Center, Salk Institute for Biological StudiesLa Jolla, CA, USA

**Keywords:** linker for activation of T cells, membrane domain, plasma membrane, signal transduction, super-resolution microscopy, T cell activation, T cell receptor

## Abstract

Genetic and biochemical studies have identified a large number of molecules involved in T cell signaling. They have provided us with a comprehensive understanding of protein–protein interactions and protein modifications that take place upon antigen recognition. Diffraction limited fluorescence microscopy has been used to study the distribution of signaling molecules on a cellular level. Specifically, the discovery of microclusters and the immunological synapse demonstrates that T cell signaling cascades utilizes spatial association and segregation. Recent advancements in live cell imaging have allowed us to visualize the spatio-temporal mechanisms of T cell signaling at nanometer scale resolution. This led to the discovery that proteins are organized in distinct membrane domains prior and during T cell activation. Evidently, plasma membrane structures and signaling molecule distributions at all length scales (molecular to cellular) are intrinsic to the mechanisms that govern signaling initiation, transduction, and inhibition. Here we provide an overview of possible plasma membrane models, molecular assemblies that have been described to date, how they can be visualized and how they might contribute to T cell signaling.

## PLASMA MEMBRANE MODELS

Spatio-temporal signal control in T cells is closely linked to the plasma membrane structure. Despite extensive studies over the past four decades, a comprehensive theory of the plasma membrane has continued to elude us. Here, we describe three models, which are not mutually exclusive, that can explain reduced diffusion rates and/or the non-equilibrium and heterogeneous distribution of proteins and lipids. It is not our intention to favor or exclude any membrane models and, based on our current knowledge, alternative models for the architecture of the plasma membrane are equally possible.

### “LIPID RAFT MODEL” (Simons and van Meer, 1988; Simons and Sampaio, 2011)

**FIGURE 1:**
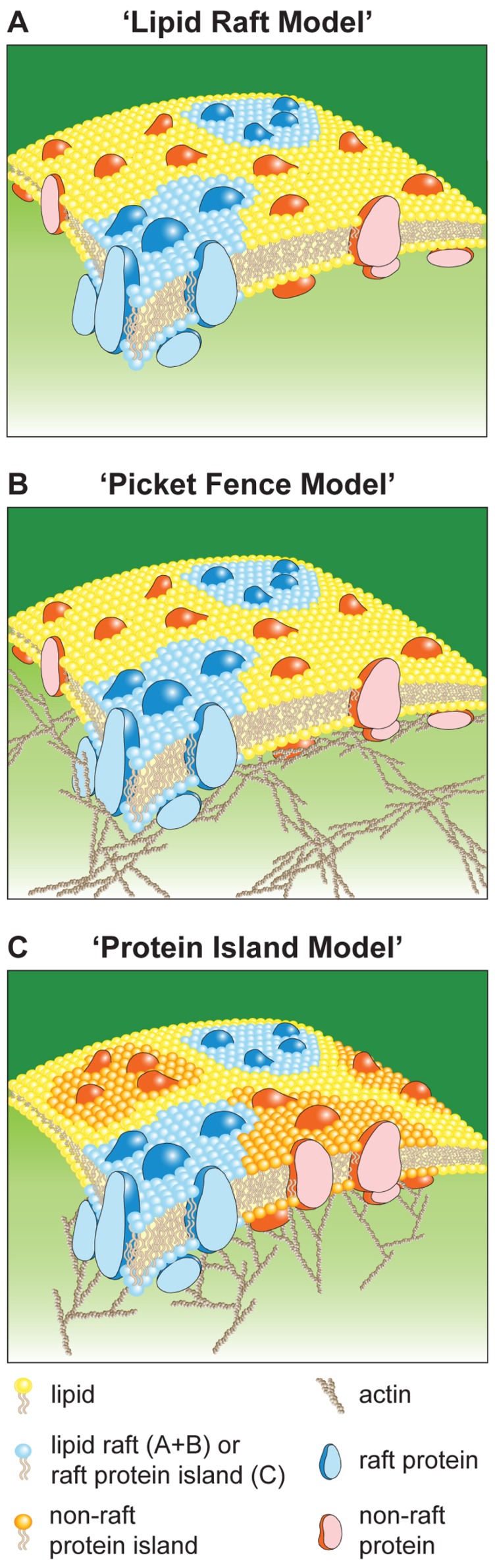
**Schematic of plasma membrane models.** Lipid Raft Model **(A)**, Picket Fence Model **(B)**, and Protein Island Model **(C)**.

This model (**Figure 1A**) is based on biophysical, microscopy, and biochemical studies. It proposes that, at any given time, approximately 35% of all membrane proteins are localized into membrane domains termed lipid rafts (Levental et al., 2010). The remaining proteins (65%) are randomly distributed and can move “freely” in accordance with the original fluid mosaic model by Singer and Nicolson (1972). The current view is that lipid rafts are dynamic nanoscale assemblies enriched for sterols and sphingolipids. Lipid rafts can be stabilized and enlarged through specific lipid–lipid, lipid–protein, and protein–protein interactions. Specifically interactions with cellular scaffolds, such as the actin cytoskeleton, have been shown to stabilize and enlarge lipid rafts (Viola and Gupta, 2007). Post-translational modifications (e.g., GPI-anchors or palmitoylation) can localize proteins into lipid rafts. A wide range of dimensions for lipid rafts have been reported using a variety of techniques, e.g., ~10 nm by fluorescence resonance energy transfer (FRET; Goswami et al., 2008); 12–24 nm (Prior and Hancock, 2012), 30–700 nm (Lillemeier et al., 2006), and 100–150 nm (Cambi et al., 2006) by electron microscopy; <20 nm by stimulated emission depletion (STED) microscopy (Eggeling et al., 2009); 100–200 nm by pair-correlation photo-activated localization microscopy (PALM; Sengupta et al., 2011); <120 nm variable spot size fluorescence correlation spectroscopy (FCS; Lenne et al., 2006). The same variability has been seen for the life-time of lipid rafts spanning from milliseconds (Eggeling et al., 2009) to seconds (Brameshuber et al., 2010), and minutes if stabilized through coalescence as seen for T cell microcluster (MC; Bunnell et al., 2002; Campi et al., 2005). The broad range of dimensions and life-times might be due to differences in detection methods or the existence of different lipid raft types (Kenworthy, 2002; Zacharias et al., 2002; Wilson et al., 2004).

### “PICKET FENCE MODEL” (Kusumi et al., 2005)

In comparison to artificial membranes, diffusion in the plasma membrane of living cells is 10- to 100-fold reduced (Murase et al., 2004). This together with single particle and molecule tracking data led to the “picket fence model” (**Figure 1B**). This model suggests that the plasma membrane is divided into “confinement zones” in which molecules are temporarily retained. Confinement is cholesterol independent and depolymerization of actin increases the dimensions of confinement zones. These results were interpreted such that confinement zones are formed through non-specific diffusion barriers of trans-membrane proteins with their immediate lipid environment (pickets) and cytoskeletal filaments that are located close and parallel to the plasma membrane (fence). Molecules undergo “hop-diffusion” when they break through the fence into a neighboring zone. Therefore, molecules show unrestricted diffusion within confinement zones and reduced mobility on a cellular level. Confinement zones are 30–250 nm in diameter and hop-diffusion occurs every 1–25 ms or 3 ms to 1 s for lipids or trans-membrane proteins, respectively (Kusumi et al., 2004). Because confinement is unspecific and confinement zones cover the entire plasma membrane with shared borders they have no effect on protein distributions. Lipid rafts can co-exist in this model and their mobility and stability is likely to be affected by the diffusion barriers.

### “PROTEIN ISLAND MODEL” (Lillemeier et al., 2006, 2010)

This model (**Figure 1C**) is based on electron and super-resolution microscopy. It proposes that all plasma membrane proteins (100%) are segregated into distinct membrane domains, termed protein islands (PIs), according to their function and nature. These domains are enriched for cholesterol and associated with the actin cytoskeleton, which regulates their positioning, separation, and/or coalescence. In this model lipid rafts are a subpopulation of PIs, which can be divided into non-raft and raft based on the characteristics of the molecules associated with them. “Protein-free” and cholesterol-low membrane regions can separate PIs. Here, “hop-diffusion” is postulated to be the translocation of a molecule from one PI to another via temporarily shared borders. Thus, transient confinement is due to localization of proteins into PIs. The diffusion behavior of molecules is identical to that in the picket fence model, unrestricted movement within PIs and restricted diffusion on a cellular level.

## MOLECULE ARRANGEMENTS AND COMPARTMENTALIZATION

Many terms are used to describe molecular arrangements and cellular compartments that are involved in the spatio-temporal control of T cell signaling. Here we define and describe some of them, importantly, others might use them in different contexts. In addition, this section aims to raise awareness of possible controversies and misunderstandings that are caused by ambiguous nomenclature.

### MONOMER

The term monomer is used to describe a single molecule or complex (e.g., T cell receptor, TCR) that moves within the plasma membrane without active or specific restriction of its mobility by other molecules.

### DIMER AND OLIGOMER

A homo- or hetero-dimer consists of two interacting molecules of the same or different type, respectively. Oligomers (a.k.a. multimers) formed by interacting molecules of numbers higher than two and are often called a complex as well.

### CLUSTER

Cluster describes the assembly of few or many molecules. Clusters fall into at least two categories: “stable” and “dynamic.” This difference can have dramatic effects on the interpretation of data and the molecular mechanisms in place. If a cluster is stable, it is a static association of molecules that functions independent of its environment. A dynamic cluster constantly exchanges with its surroundings and maintains equilibrium of association and dissociation. A dynamic cluster can form based on temporary interactions between its content or on an increased likelihood of molecules to localize to a specific area. The later could be the definition of a membrane domain.

### MEMBRANE DOMAIN

An area within a cellular membrane, most often the plasma membrane, in which specific lipids and proteins are enriched. Membrane domains could be either “fluid” or “static”. Within fluid membrane domains molecules or complexes can move in unrestricted fashion within the membrane domain. It is not clear how borders and dimensions of fluid membrane domains are established and maintained. Domains might have stable borders based on lipid phase separation or physical barriers that retain molecules within them. Alternatively, attractive forces between molecules and the quantity of molecules might determine the stability and dimensions of domains. Proteins within static membrane domains do not move freely for the time the domain exists. Such a membrane domain would be similar to a cluster that includes lipids.

### DETERGENT RESISTANT MEMBRANE

The term detergent resistant membranes (DRMs; Yu et al., 1973; Brown and Rose, 1992) and lipid rafts are often used indiscriminately. However, comprehensive studies have shown that these terms do not necessarily describe the same structures. DRMs are relative to a well-defined experimental procedure. Specifically, they are membrane structures that resist cold detergent extraction and float in sucrose gradients (Yu et al., 1973; Edidin, 2003). Detergent extraction is controversial and several short-comings have been described, such as differences in the solubility of the outer and inner membrane leaflets, temperature-induced changes in the lipid organization, formation of holes leading to mixing of lipids from the inner and outer leaflets, promotion of the liquid ordered phase, and therefore DRMs, by detergent (e.g., Triton X-100; Munro, 2003). Thus, “detergent resistant membranes should not be assumed to resemble biological lipid rafts in size, structure, composition, or even existence” (Heerklotz, 2002).

### LIPID RAFT

The concept of lipid rafts (Simons and van Meer, 1988; described in the first section of the review) is based on a molecular interpretation of lipid and protein characteristics. Often lipid rafts are studied *in vivo* by cholesterol extraction, which can induce dramatic off-target effects. For example, cholesterol depletion causes cell-death and increases the membrane permeability for ions (Munro, 2003). This has been reported in T cell signaling, where tyrosine phosphorylation was induced upon ligand binding to cholesterol-depleted cells, but the typical Ca-influx was prohibited (Pizzo et al., 2002). Lipid rafts are often detected using cholera-toxin B-subunit labeling of ganglioside GM1. However, cross-linking of GM1 causes increased endocytosis, which might be detected as induced clustering. Moreover, lipid rafts and phase separation, thought to be a basic principle of lipid raft formation, are commonly studied in synthetic membrane systems, which are less complex and often studied below physiological temperatures. Visualization of lipid rafts with novel dynamic, high-speed, and super-resolution techniques has (see first section) and will allow more definite descriptions of lipid rafts.

### NANOCLUSTER

Nanoclusters (NCs) have been described for integrins and signaling molecules such as ras, FcR, and TCR (Detmers et al., 1987; Wilson et al., 2000; Prior et al., 2003; Cambi et al., 2006; Schamel et al., 2005; Sherman et al., 2011). NCs have been visualized mainly by electron microscopy and just recently by fluorescent super-resolution microscopy. They have diameters of 12–150 nm and are thought to be formed through protein–protein interactions. They are most commonly associated with lipid raft proteins, however, several types of ras molecules form NCs in a cholesterol-independent manner (Prior and Hancock, 2012). Due to the extensive sample manipulation in electron microscopy, these structures have often been described as experimental artifacts. However, more recently clustering of many molecules has been confirmed by fluorescent super-resolution and dynamic microscopy.

### PROTEIN ISLAND

Protein islands (Lillemeier et al., 2006, 2010) are structures (40–250 nm wide) in which signaling molecules are organized prior to T cell activation (see first section for PI model). The same structures have been described later as NCs (Sherman et al., 2011). A possible distinction between PIs and NCs is their postulated origin. NCs are based on the idea that signaling molecules, at least partially, form complexes prior to ligand engagement. PIs are thought to be membrane domains with an environment that attracts specific proteins. In contrast to NCs, molecules can move freely within and exchange between PIs.

### TRANSIENT CONFINEMENT ZONE

Transient confinement zones (TCZs; Simson et al., 1995; Sheets et al., 1997; Dietrich et al., 2002) have been observed by single molecule or particle tracking. TCZs are membrane regions where molecules are trapped and their diffusion is substantially slowed (diffusion rates are ~50% of non-confined molecules). TCZs are 200–300 nm wide and molecules are typically trapped for 5–10 s. Often molecules are trapped again after several seconds of random and fast movement. Transient confinement is mostly cholesterol dependent and, thus, has often been linked to the association of proteins to lipid rafts. Transient confinement of the T cell signaling molecule linker for activation of T cells (LAT) to MCs has been observed by single molecule tracking (Douglass and Vale, 2005). Transient confinement has been redefined as stimulation-induced temporary arrest of lateral diffusion (STALL) to include the possibility of actin binding rather than trapping into zones (Suzuki et al., 2007). Importantly, TCZs are distinct from confinement zones described earlier for the picket fence model. TCZs do not cover the entire cell surface and confinement is at a different time scale (seconds versus milliseconds).

### MICROCLUSTER 

Initial T cell signal transduction takes place mostly in MCs (Bunnell et al., 2002; Campi et al., 2005), which are 200–1000 nm wide. They form within seconds of ligand binding and move to the center of the T cell–antigen presenting cell (APC) contact site in an actin- and microtubule-dependent manner. MCs contain most molecules of the TCR and CD28 signaling cascades. The organization of the molecules within them remains controversial. PIs/NCs could be stable over the course of T cell activation and become MC subunits. Alternatively, the PIs/NCs could fuse after MC formation and their content mix. MCs have often been described as stabilized and enlarged lipid rafts (Viola and Gupta, 2007).

### SUBSYNAPTIC VESICLE

Subsynaptic vesicles (SSVs; Bonello et al., 2004; Purbhoo et al., 2010) are mostly endosomes (~70%) or originate from the Golgi (~22%) based on Rab7 and Rab8a staining, respectively. The vesicles translocate to the T cell–APC interface upon antigen recognition and repeatedly interact with MCs at the plasma membrane. To date only LAT has been associated with these vesicles.

### IMMUNOLOGICAL SYNAPSE

The immunological synapse (IS; Monks et al., 1998; Grakoui et al., 1999) is several micrometer wide and forms within minutes at the contact site between T cell and APC through MC translocation and accumulation. Originally a “mature” IS has been described as a bull’s eye pattern with signaling molecules in the center and adhesion molecules in the periphery. Comprehensive studies of the IS have shown that it can take different shapes and sizes dependent on ligand concentration and the ratio of activating and co-stimulatory signals. Distinct regions within the IS are described as supramolecular activation clusters (SMACs; see below).

### SUPRAMOLECULAR ACTIVATION CLUSTER

The IS is divided into sub-regions called supramolecular activation clusters (SMACs; Monks et al., 1998). Namely the central-SMAC (c-SMAC), peripheral-SMAC (p-SMAC), and distal-SMAC (d-SMAC). Each region contains a specific subset of T cell signaling molecules. Depending on the experimental conditions, signaling molecules are often reported to localize to more than one region over the course of T cell activation. Recently, it has become clear that at least the c-SMACs can be subdivided further into regions that contain CD28 and little TCR (TCR^low^) versus regions that contain more TCR but no CD28 (TCR^high^; Saito et al., 2010).

## IMAGING TECHNIQUES TO VISUALIZE T CELL SIGNALING

Here we introduce some of the latest imaging techniques that have been, and will be, essential for a comprehensive understanding of the spatio-temporal control of T cell activation and the plasma membrane in general.

### BINDING SURFACES

Functionalized surfaces are frequently used to orientate and immobilize T cells. Non-activating surfaces use unspecific ligands (e.g., poly-L-lysine), adhesion molecules (e.g., leukocyte function-associated antigen, LFA-1), or antibodies against surface molecules that do not activate T cells [e.g., anti-major histocompatibility complex (MHC) class I]. Activating surfaces present either antibodies that cross-link the TCR and CD28, or recombinant ligands that activate T cells through binding to the TCR, CD28, and LFA-1. More physiological conditions are achieved through binding of T cell ligands to fluid glass-supported lipid bilayers, which renders the ligands mobile. This allows the T cell signaling molecules to organize themselves without any interference from the surface bound ligands. The disadvantage of any glass surfaces is their rigidity, which inhibits plasma membrane protrusions and invaginations, and influences cytoskeletal rearrangements. These surfaces are widely used in T cell studies and the same principles are applied to other cell types.

### OPTICAL TWEEZERS

Optical tweezers (a.k.a. optical trap) are a highly focused laser beam that can be used to trap and manipulate small dielectric particles (Ashkin et al., 1986). The radiation pressure in the laser beam waist applies attractive or repulsive forces in the order of piconewtons. This technology has been used to position T cell-APC couples and orientate their IS in the imaging plan of a confocal microscope (Oddos et al., 2008). This technology allows faster image acquisition and provides the most physiological conditions.

### TOTAL INTERNAL REFLECTION MICROSCOPY

In Total Internal Reflection Microscopy (TIRFM; Axelrod, 1981) a laser beam that is reflected on the glass–water interface of a specimen causes an evanescent wave that penetrates less than 200 nm into the sample to excite fluorophores. This is ideal to image plasma membrane structures with reduced fluorescence background. Single molecule techniques rely on high signal-to-noise ratios and benefit substantially from this type of illumination.

### SUPER-RESOLUTION MICROSCOPY

Traditional fluorescence microscopy is diffraction limited and the highest possible resolution is approximately half the emission wavelength (~250 nm). Super-resolution techniques can achieve resolutions between 10 and 100 nm and have been reviewed previously (Kasuboski et al., 2012). They are either based on (i) single molecule detection [PALM; Betzig et al., 2006 and stochastic optical reconstruction microscopy (STORM); Rust et al., 2006]; (ii) reduced illumination volumes (STED microscopy; Hell and Wichmann, 1994); or (iii) illumination with periodic patterns [structured illumination microscopy (SIM); Bailey et al., 1993].

### FLUORESCENCE CORRELATION SPECTROSCOPY

Fluorescence correlation spectroscopy (FCS; Magde et al., 1974; Fitzpatrick and Lillemeier, 2011) measures the fluctuations in fluorescence intensity within a small observation area. The fluctuations in fluorescence intensity are caused by the exchange of fluorophores between the illuminated area and its surroundings. Auto-correlation analyses of single fluorophore fluctuations provide particle concentrations and diffusion rates. Fluorescence cross-correlation spectroscopy (FCCS) measures fluctuations from two fluorophores and determines their rate of co-movement.

### TWO-COLOR COINCIDENCE DETECTION MICROSCOPY

Two-color coincidence detection (TCCD; James et al., 2007) determines the frequency at which two differently labeled molecules are in the same diffraction limited excitation volume. Molecules that interact or are located within same membrane structures show increased coincidence detection. This method uses extremely low labeling efficiency to detect and time-resolve single molecule events. Thus, this method has low sensitivity and is generally used in comparative analyses.

### FLUORESCENT SPECKLE MICROSCOPY

Fluorescent speckle microscopy (FSM; Salmon and Waterman, 2011) can be used to detect the movement and the assembly dynamics of cellular structures (e.g., actin and microtubule). FSM is based on very low labeling, approximately 0.5% of a specific molecule species with approximately 1–10 fluorophores per diffraction limited volume. Using low background detection and photo-bleaching structures appear “speckled.” Translation of speckle distribution indicates movement of structures and changes in intensity reveal assembly dynamics and subunit turnover.

### MEMBRANE SHEET TRANSMISSION ELECTRON MICROSCOPY

The basic principle of the technique is that cells are broken open (“ripped”) through mechanical forces applied by the separation of two opposing surfaces sandwiching the cell, with one of the surfaces placed on an EM grid and visualized with transmission electron microscopy (TEM; Sanan and Anderson, 1991; Wilson et al., 2000; Lillemeier et al., 2006; Lillemeier and Davis, 2011). Staining of lipids with heavy metals has shown that the lipid bilayer continuity is maintained in this procedure and membrane sheets without holes can be obtained (Lillemeier et al., 2006). Specific proteins on the inner membrane surfaces can then be detected with gold conjugated probes. This technique has the highest resolution, but requires fixation and extensive sample manipulation, both often associated with cluster artifacts.

## T CELL SIGNAL TRANSDUCTION

T cell signal transduction has been extensively reviewed (Smith-Garvin et al., 2009). Thus, we only give a brief overview of key events that are necessary to understand the correlation between signaling and spatial distributions described in the following section.

T cells require two distinct activation signals by APCs for proliferation, differentiation, and function. The first signal is through the specific recognition of peptide–MHCs by the TCR and its co-receptor (CD4 or CD8). The importance of the co-receptor signal becomes more alleviated when TCR affinity is very strong and/or ligand concentration is low. The second signal is through another family of co-receptors that includes the co-stimulatory CD28 and co-inhibitory cytotoxic T-lymphocyte antigen-4 (CTLA-4). CD28 and CTLA-4 share their ligands CD80 (B7-1) and CD86 (B7-2) expressed on APCs. The balance and nature of co-stimulatory and co-inhibitory signals can amplify or weaken a T cell response to TCR ligation, possibly leading to auto-immunity or non-responsiveness (“anergy”). In addition, adhesion molecules mediate contacts between APCs and T cells and may also influence T cell signaling. Often these interactions are utilized independently of TCR engagement for migration and the search for potential APCs. Examples for proteins with these functions are the integrin LFA-1 and CD2, respectively.

T cell receptor engagement activates a tyrosine kinase signaling cascade. The first kinase in the TCR signaling cascade is the membrane-bound leukocyte-specific protein tyrosine kinase (LCK), which exist in a “free” and a co-receptor (CD4 or CD8) bound form. Co-receptor association aids LCK recruitment to the ligand-engaged TCR and its activation by trans- and auto-phosphorylation. Subsequently, LCK phosphorylates the immunoreceptor tyrosine-based activation motifs (ITAMs) of the TCR. Phosphorylated ITAMs recruit the zeta-associated protein kinase of 70 kDa (ZAP-70) to the TCR via its SH2-domains. TCR bound ZAP-70 is activated by both LCK and auto-phosphorylation. Active ZAP-70 then phosphorylates its substrates including the adaptor proteins SH2 domain-containing leukocyte protein of 76 kDa (SLP-76) and LAT. These adaptors transfer the signals onto multiple pathways, leading to T cell activation and function.

## SPATIO-TEMPORAL CONTROL OF T CELL SIGNALING

In this section, we summarize recent findings on the spatial distributions of signaling molecules using TCR, LAT, CD28, and CTLA-4 as key examples. Different activation stages of T cells are described. Specifically, we focus on quiescent T cells, early signaling events, the mature and the late IS. The spatio-temporal control of plasma membrane signaling is an evolving field and alternative interpretation and conclusion are possible and should be considered in future studies.

### QUIESCENT T CELL (Figure 2A)

Based on biochemical data, it has been proposed that T cell signaling molecules can be divided into non-raft and raft. Specifically, TCRs are not found in DRMs while LAT, LCK, and CD4/CD8 are detected in DRMs (Zhang et al., 1998; Harder and Kuhn, 2000). This was supported by the sensitivity of TCR signaling to cholesterol depletion. Using scanning electron microscopy and native polyacrylamide gel electrophoresis Alarcón and colleagues found that a significant portion of the TCR exist as oligomers and form multivalent receptor complexes (Schamel et al., 2005). Using EM, super-resolution microscopy, and FCCS in membrane sheet and live T cells, we have shown that all TCR and LAT molecules are pre-organized in distinct PIs/NCs while maintaining their overall mobility (Lillemeier et al., 2010). Segregation of signaling molecules into distinct PIs/NCs could insulate signaling components from each other and contribute to uphold a quiescent state. Simultaneously, arranging the signaling cascades into “building blocks” with inherent affinity for each other keeps the T cell on the edge of activation similar to a “loaded gun”. Pre-organization of T cell signaling molecules was confirmed by other super-resolution studies (Sherman et al., 2011; Williamson et al., 2011). In contrast to our studies, over 50% of co-localization for TCR and LAT was observed. Additionally, earlier studies have shown that LAT exists in plasma membrane and vesicular pools, later described as subsynaptic vesicles (SSVs; Bonello et al., 2004; Purbhoo et al., 2010). Both pools are maintained through a balance of endo- and exocytosis.

**FIGURE 2:**
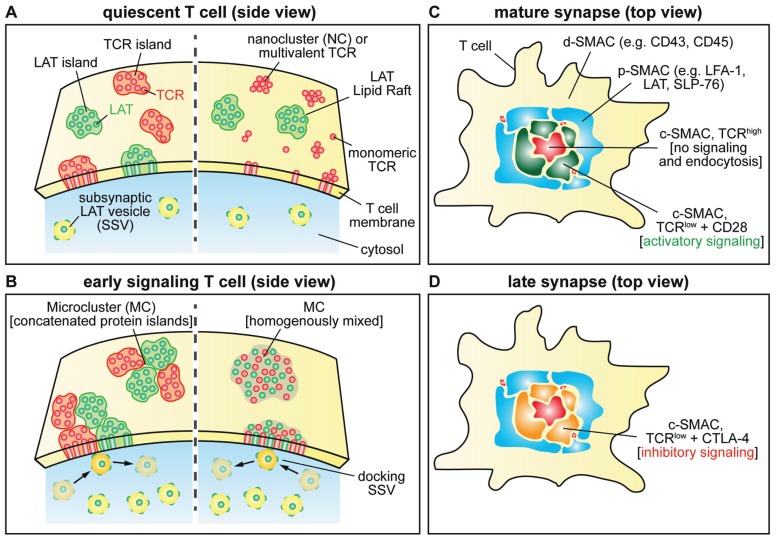
**Schematic of signal molecule distributions during different stages of T cell activation.** **(A)** This panel shows the distribution of TCR and LAT molecule in protein islands (left) and the multivalent TCR or nanocluster with LAT localized to lipid rafts on the right. Small numbers of SSVs are shown close to the plasma membrane. **(B)** MC formation based on protein island/nanocluster concatenation (left) and fusion (right). Accumulation of SSVs below the T cell–APC contact site. SSVs repeatedly interact with MCs. **(C)** Molecular arrangement in the mature IS. Examples for molecules localized in the different SMACs are shown. The c-SMAC is divided in two zones depending on TCR concentration and CD28 localization (TCR^low^) and TCR^high^). **(D)** CTLA-4 replaces CD28 in the TCR^low^ zones inhibiting T cell signaling in the late IS.

### EARLY T CELL SIGNALING (Figure 2B)

Upon antigen recognition dramatic reorganization of signaling molecules takes place. Biochemical experiments found that the TCR and other signaling molecules associate with DRMs after T cell activation. These experiments suggested that the TCR signaling cascade is assembled in lipid rafts (Viola and Gupta, 2007). Using fluorescence microscopy, Samelson and colleagues showed that T cell signaling molecules assemble to form MCs (Bunnell et al., 2002), which have been suggested to be large lipid rafts. MC formation has been studied extensively and initial T cell signaling has been associated with them. MC formation is actin dependent and takes place even in the mature IS (Campi et al., 2005). MCs move toward the center of the IS along microtubules using dynein motors (Hashimoto-Tane et al., 2011). Super-resolution studies of signaling molecules have shown that MCs are formed through association of pre-formed PIs or NCs (Lillemeier et al., 2010; Sherman et al., 2011). These findings have far-reaching implications for our view of T cell signaling. It can explain the sensitivity of T cells and the short time required to assemble the membrane-associated signaling cascades. PIs/NCs are likely to be the smallest activation unit and thus TCRs become recruited into MCs in a ligand-independent way as shown by Dustin and colleagues (Varma et al., 2006). This could be a crucial principle enabling co-activation with endogenous peptide/MHC (Wulfing et al., 2002) and serial triggering of TCRs (Valitutti et al., 1995), an old concept of T cell activation. If PIs/NCs remain intact within MCs is an ongoing debate. Our studies of TCR and LAT show that MCs are concatenated PIs/NCs that remain distinct and do not exchange their content (Lillemeier et al., 2010). This suggests that the spatial segregation of signaling molecules is an inherent part of signal transduction mechanisms in the plasma membrane. If confirmed, future studies will have to determine how a spatially segregated signal transduction cascade can complete the identified sequence of protein interactions and modifications. Interestingly, TCR association with lipid rafts might not be due to its relocation, but due to coalescence of non-raft and raft domains in MCs. However, super-resolution studies from the Samelson group suggest that formation MC leads to different degrees of mixing for signaling molecules, specifically ~50% for TCR + LAT, ~100% for TCR + ZAP-70, and ~20% for LAT + ZAP-70 (Sherman et al., 2011). This study also describes that signaling molecules, specifically SLP-76, can associate at the rim of MCs. If mixing of signaling molecules takes place in MC, signal transduction can be achieved through random contacts. A major difference between these studies is that ours used primary mouse T cells bound to non-activating and activating glass-supported lipid bilayers, while Samelson’s used Jurkat T cells on immobilized antibody surfaces. Additional super-resolution studies will have to be conducted to further elucidate the substructures of MCs. The group of Davis has observed that LAT containing SSVs accumulate underneath the T cell–APC contact site upon TCR activation (Purbhoo et al., 2010). Motile SSVs repeatedly moved to MCs, where they were retained for short periods of time. LAT phosphorylation was greatest in MCs that had a recent interaction with SSVs. Gaus and colleagues took this concept a step further and suggest that only SSVs contain active LAT, while plasma membrane-associated LAT is not involved in signaling at all (Williamson et al., 2011). This implies that LAT is recruited from SSVs to the plasma membrane in *trans*. Additional studies are necessary to distinguish between the different models for LAT activation and to what degree they take place simultaneously.

### MATURE IMMUNOLOGICAL SYNAPSE (Figure 2C)

Over a period of 5–10 min after antigen recognition a mature IS forms through actin- and microtubuli-dependent transport of MCs to the center of the T cell–APC contact site (Campi et al., 2005; Hashimoto-Tane et al., 2011). MCs fall apart when they reach the center of the T cell–APC contact site and their “cargo” either enters the c-SMAC (e.g., TCR and CD28), while other molecules remain in the p-SMAC (e.g., LAT and SPL-76). If the MCs contain distinct PIs/NCs, this process would only require their dissociation. The finding that TCR PIs/NCs exist within the c-SMAC supports this (Lillemeier et al., 2010). Based on the distribution of TCR and CD28, the c-SMAC can be divided into two regions. TCR^high^ regions contain no CD28 and show little or no signaling activity (Saito et al., 2010). These regions are rigid and are most likely areas of TCR endocytosis. TCR^low^ regions contain CD28 and have active signaling complexes, thus referred to as signaling c-SMAC. These are dynamic areas with high exchange rates for signaling molecules. Stronger TCR and weaker CD28 activation increases the size of TCR^high^ regions, and the opposite increases the size of TCR^low^ regions.

### LATE IMMUNOLOGICAL SYNAPSE (Figure 2D)

In this stage, T cell signaling is reduced while the T cell–APC contact is maintained. Prior to activation, the majority of CTLA-4 is located in the *trans*-Golgi network (TGN), endosomes, and lysosomes (Valk et al., 2006). Upon formation of a mature IS, CTLA-4 directly accumulates in the c-SMAC, which leads to downregulation of CD28 signaling and its endocytosis (Rudd et al., 2009). One of the major CTLA-4 mechanisms suggested is the competition with CD28 for CD80 and CD86. Due to bridging of ligand dimers, CTLA-4 has much higher affinity to the ligands (~12 nM) than CD28 (~200 nM).

## SUMMARY

It has become clear that spatio-temporal mechanisms on all length scales are crucial prior and during any stage of T cell activation. Here, we have described current views of the plasma membrane architecture, recent findings on membrane compartmentalization in T cells, and how they affect our thinking about signal transduction in T cells. These new insight would not have been possible without recent advances in imaging techniques. It is now possible to visualize molecular events and study them in their physiological environment. More sophisticated imaging techniques and analysis tools have to be developed to obtain a comprehensive and conclusive understanding of how plasma membrane signaling is organized in space and time.

## Conflict of Interest Statement

The authors declare that the research was conducted in the absence of any commercial or financial relationships that could be construed as a potential conflict of interest.
